# Living Conditions and the Mental Health and Well-being of Refugees: Evidence from a Large-Scale German Survey

**DOI:** 10.1007/s10903-019-00968-5

**Published:** 2020-01-24

**Authors:** Lena Walther, Lukas M. Fuchs, Jürgen Schupp, Christian von Scheve

**Affiliations:** 1 Clinic for Psychiatry and Psychotherapy, Charité – University Medicine Berlin, Hindenburgdamm 30, 12203 Berlin, Germany; 2 Institute of Sociology, Freie Universität Berlin, Berlin, Germany; 3 German Institute for Economic Research, Socioeconomic Panel, Berlin, Germany

**Keywords:** Refugees, Mental health, Well-being, Integration

## Abstract

Refugees are at an increased risk of mental health problems and low subjective well-being. Living circumstances in the host country are thought to play a vital role in shaping these health outcomes, which, in turn, are prerequisites for successful integration. Using data from a representative survey of 4325 adult refugees who arrived in Germany between 2013 and 2016, we investigated how different living conditions, especially those subject to integration policies, are associated with psychological distress and life satisfaction using linear regression models. Our findings show that an uncertain legal status, separation from family, and living in refugee housing facilities are related to higher levels of distress and decreased life satisfaction. Being employed, contact to members of the host society, and better host country language skills, by contrast, are related to reduced distress and higher levels of life satisfaction. These associations should inform decision making in a highly contested policy area.

**Electronic supplementary material:**

The online version of this article (10.1007/s10903-019-00968-5) contains supplementary material, which is available to authorized users.

## Introduction

Research has consistently shown that refugees are at a particular risk of facing mental health problems (reviewed in [1–5]). Despite a substantial between-study heterogeneity in refugees’ mental illness prevalence rates, forced migration has persistently been linked to increased rates of mental illnesses, chiefly, post-traumatic stress disorder (PTSD), depression, and anxiety disorder [5–7]. Even considering that those who embark on flight are likely to exhibit resilience (‘Healthy Immigrant Effect’ [8]), refugees are particularly at risk of facing psychological distress as sequelae of traumatic or stressful experiences before or during flight [4, 9, 10].

However, studies also indicate that the refugee mental health burden has roots beyond discrete traumatic experiences or the experience of displacement. A review of studies on refugee mental health and its predictors shows that the psychological burden of the refugee experience is substantially elevated even when refugee mental health is compared to the mental health of other groups exposed to war and violence [11].

Studies based on large-scale survey data have also shown substantially lower levels of overall subjective well-being amongst immigrant populations compared to natives [12, 13]. Even when migration leads to economic prosperity, it may remain associated with lower levels of well-being [14, 15].

Importantly, well-being and mental health are not just outcomes of past experiences, but also of present social, cultural, and economic circumstances [16]. While research on the effects of pre-migration stressors on mental health dominates the literature, post-migration stressors seem to have an equally substantial impact. In addition to migration-related acculturative stress (see [17–19]), factors associated with refugees’ mental health and well-being include uncertainty related to legal proceedings, detainment in refugee camps, discrimination, social isolation, financial problems, unemployment, separation from family, safety concerns, and uncertainty about the country of origin’s future (reviewed in [5, 10, 11, 20]). Further studies show that the subjective well-being of migrants in general is associated with host country language proficiency and identification [21] and that it is linked to the quality of public goods, the climate of immigrant reception, and the extent of economic inequality after migration [22].

Some of these post-migration stressors are directly affected by integration policies and measures in a hosting country. Since successful integration depends on mental health and well-being as vital personal resources [20, 23], what is at stake is the prevention of a vicious cycle between poor mental health as a consequence of traumatic experiences and post-migration stress, functional impairments, and the exacerbation of post-migration stressors.

The present study therefore investigates how *psychological distress* (comprising the most prevalent symptoms of poor mental health) and *life satisfaction* (the cognitive dimension of subjective well-being) of recently arrived refugees in Germany are associated with integration measures aimed at promoting integration and with other, more general post-migration living conditions. Controlling for socio-demographics and pre- as well as peri-migration stressors, we model psychological distress and life satisfaction as functions of (a) the outcome of the asylum process, (b) seeking family reunification, (c) type of housing, (d) being in education, (e) being employed, (f) attendance of integration and language courses, (g) time spent with co-nationals, with German nationals, and with persons from other countries, and (h) German language ability.

## Methods

### Data and Participants

The data used in this study comes from the first wave (2016) of the IAB-BAMF-SOEP dataset, an annual, representative survey of 4465 adults (at least 18 years of age), predominantly refugees and asylum seekers who arrived in Germany between January 1, 2013 and January 31, 2016 (see [24, 25] for details). Respondents completed the survey in computer-assisted face-to-face interviews by trained interviewers using audio files in five different languages. Participation was voluntary.

We excluded 21 respondents from our analyses due to missing corresponding household interviews. A further 27 respondents were excluded on the basis that they were mandated to leave Germany within the coming month. In these cases, self-reported measures of mental health and well-being are unlikely to reflect the integration measures and living conditions we are interested in evaluating. We excluded 92 further respondents from our analysis on the basis that they were members of the sampled asylum seekers’ households who were not themselves refugees who had arrived in Germany between 2013 and 2016, resulting in an analysis sample size of 4,325 respondents.

### Measures

#### Dependent Variables

##### Psychological Distress

To measure psychological distress, we used the *Patient Health Questionnaire for Depression and Anxiety* (PHQ-4), a very brief and well-validated measurement instrument [26–28]. This 4-item battery uses a 4-point Likert-type scale (scores 0–3, (0) meaning symptoms not at all experienced in past 2 weeks, (1) on several days, (2) on more than half the days, (3) nearly every day) to screen for the core symptoms of depression (depressed mood, anhedonia) and anxiety (uncontrollable worrying and feeling nervous) with two separate scores or to yield a single overall measure of the degree of psychological distress ranging from 0 (no distress) to 12 (severe distress) [26, 29]. We used the total score of the PHQ-4, measuring psychological distress characterized by symptoms of depression and anxiety, in order to capture the complete spectrum of variance [29]. Despite its brevity, the PHQ-4 performs very similarly to the combined longer PHQ-8 and the GAD-7 [26], which, in turn, are well-established as excellent screening tools for depression and anxiety, respectively [30, 31]. Previous studies have shown that the two depression items in the PHQ-4 match outcomes of the DSM-IV Structured Clinical Interview with a sensitivity of 87% and a specificity of 78% for major depressive disorder [32]. The two anxiety items perform very well at diagnosing generalized anxiety disorder (Area Under the Curve (AUC) = 0.91), panic disorder (AUC = 0.85), social anxiety disorder (AUC = 0.83), and PTSD (AUC = 0.8) [26]. In another sample, the PHQ-4 diagnosed depression and anxiety disorders with AUCs of 0.84 and 0.79 [28]. The PHQ-4 also shows good internal reliability with Cronbach’s alphas of 0.79 for a Tanzanian [33], 0.84 for a Colombian [34], and 0.78 for a German sample [27]. In our sample, the internal consistency of the scale was equally acceptable (Cronbach's alpha = 0.77).

##### Life Satisfaction

We assessed life satisfaction, understood as the cognitive-evaluative dimension of subjective well-being, using a standard single-item measure widely applied in large national surveys where the costs of administering more comprehensive multi-item scales are prohibitive [35–37]. This measure yields acceptable reliability (range of r scores: 0.68–0.74) when tested longitudinally [38], good criterion validity when compared to a well-established multi-item scale, and similar construct validity to the multi-item scale [39]. Many studies have also demonstrated high correlations between judgments of global life satisfaction and more comprehensive measures of satisfaction in key life domains [40, 41].

#### Independent Variables

##### Sociodemographic Control Variables

Levels of education were aggregated according to ISCED standards as follows: low (early childhood education, primary education, lower secondary education), medium (upper secondary, post-secondary non-tertiary education, short-cycle tertiary education), and high (bachelor’s or master’s degree or equivalent, doctoral or equivalent degree). Nationality was reduced to categories with at least 100 observations: Syrian, Afghan, Iraqi, Eritrean, Other. Time in Germany was measured in years passed between arrival in Germany and the time of the interview. Marital status was assessed with the categories ‘Married’, ‘Single’, and ‘Divorced or Widowed’, religious affiliation with the categories ‘Muslim’, ‘Christian’, ‘Other’, ‘None’.

##### Pre- and Peri-migration Control Variables

Negative flight experiences were coded ‘yes’ if any of a list of seven possible negative experiences (financial scams or exploitation, sexual assault, physical assault, shipwreck, robbery, extortion, imprisonment) was reported. They were coded ‘no’ if none of these experiences were reported and ‘wished not to report’ if the respondent chose not to answer the section on flight experiences. To count the number of distressing flight reasons, we created a numeric variable summing up the number of the following flight reasons: ‘fear of violent conflict or war’, ‘fear of military draft or forced recruitment into armed groups’, ‘persecution’, ‘discrimination’, ‘bad personal living conditions’. We did not include the following flight reasons in this index because of their lack of an obvious stressor status: ‘my family sent me’, ‘because family members left this country’, ‘because friends/acquaintances left this country’, ‘general economic situation in the country of origin’, ‘other reasons’. Finally, we created a two-level categorical variable capturing whether respondents came to Germany by themselves, combining the categories ‘arrived with family members’, ‘with friends/acquaintances’, ‘with other persons’ into one level juxtaposed with the category ‘arrived alone’.

##### Integration Measures and Post-migration Living Conditions

The legal status variable was created by combining the report of a received refugee or asylum status into one category, and counting both reports of awaiting the outcome of the initial asylum procedure and reports of awaiting the outcome of an appeal against the initial asylum procedure decision as ‘awaiting outcome’. The family reunification variable was conceived as a binary variable assigning a ‘yes’-category to reports of having either a spouse or any number of children born after 1998 and planning to bring these family members to Germany. Currently in education includes any kind of education (school, university or doctoral studies, vocational training, professional development course). Our employment status variable comprises a ‘yes’ category for any form of employment reported (full or part time, marginally employed, internships or traineeships), a ‘no’ category for a report of no current employment but seeking employment and a ‘not seeking employment’ category. Course participation was measured as the total number of courses attended out of five integration courses and general language courses. Social contacts were measured as amount of time spent with members of different communities, ranging from ‘never’ to ‘daily’. German language ability was measured as the averaged self-reported speaking, reading, and writing ability. See the SI Appendix for details.

### Analysis

All statistical analyses were conducted using R version 3.5.0 [42]. We imputed missing data in all of the variables used for analysis through multiple imputation using chained equations with the “mice” R package [43] (10 imputed datasets created, 10 iterations, seed = 41) (see SI Appendix Table A1 for missings per analysis variable). To improve the accuracy of the imputation, we used auxiliary variables selected for their theoretical relatedness to the to-be-imputed variables (see SI Appendix). Only auxiliary variables with a minimum correlation of r = 0.1 with to-be-imputed variables were used in the imputation [44].

We calculated descriptives, as shown in Appendix Tables A2 and A3, as means and standard deviations with 95%-confidence intervals or proportions with 95%-confidence intervals. The weighted values shown in the final two columns were produced using the survey weights supplied by the Socio-economic Panel of the DIW Berlin [24].

**Fig. 1:**
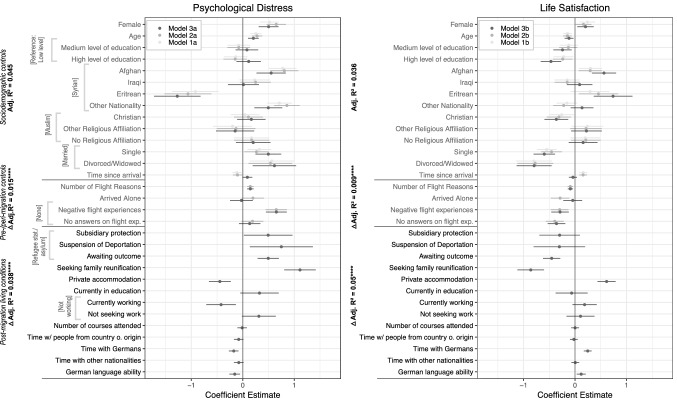
Plotted estimated regression coefficients with error bars (95% confidence intervals). Hierarchical linear regressions comprising three models each (Models 1–3a and Models 1–3b). Regression coefficient estimates pooled across 10 imputed datasets. Predictor variables are standardized for comparison purposes. Reference categories for the binary categorical predictors: gender—male, arrived in Germany alone—did not arrive alone, family reunification—not seeking reunification with a spouse or an underaged child, currently in education—currently not in education. The control variable federal state of residence was omitted from the plot for the sake of clarity. Complete numeric results for both of these models are included in the SI Appendix (Tables A4, A6). *p < .05; **p < 0.01; ***p < 0.001; ****p < 0.0001 for model comparison

In our main analysis, we calculated and pooled 10 multiple, hierarchical linear regressions to estimate associations between psychological distress, life satisfaction, and variables reflecting integration measures as well as refugees’ post-migration living conditions. The baseline models (1a, 1b in Fig. 1) predict psychological distress and life satisfaction from the sociodemographic control variables federal state of residence (not included in Figure, see SI Appendix Tables A4 and A6), age, gender, education, nationality, marital status, religious affiliation, and time since arrival in Germany. Subsequent models (2a, 2b in Fig. 1) include variables representing pre- and peri-migration stressors as further controls: the number of flight reasons, whether the respondent fled alone, and negative experiences during flight. For the full models (3a, 3b in Fig. 1), we added all key predictors (a–h) mentioned above. We did not weight our regression, but included the main factors that went into Kroh and colleagues’ [24] calculation of individual weights (gender, age, time, nationality, since arrival in Germany, legal status, and federal state of residence) as independent variables [45] (p. 57).

We assessed the statistical significance of the difference between Models 1 and 2 and Models 2 and 3, respectively, using Wald tests implemented using a function for the comparison of nested models fitted to imputed data [43, 46]. We used the same tests to confirm the joint significance of all categorical variables with significant differences between levels. Our SI Appendix includes the models using non-imputed data as robustness checks (Tables A5 and A7). A further robustness check shown in the SI Appendix (Table A8) replicates Model 3a as a proportional odds cumulative logit model using the PHQ-4 as a four-category ordered outcome (‘none’, ‘mild’, ‘moderate’, ‘severe’).

To investigate potential moderation effects between our control variables and the post-migration variables of interest, we computed interactions between key sociodemographics (gender, age, nationality, education) and each of our post-migration variables, and ran stratified regressions to examine significant interactions further. Following Chen and associates [47], we also examined possible interactions between the number of flight reasons (our best proxy for traumatic experiences in the country of origin) and post-migration living conditions in their relationship with psychological distress and life satisfaction. Because this part of the analysis is exploratory, we looked into all interactions significant at the α = 0.05 level, despite multiple comparisons (see SI Appendix for details).

## Results

### Descriptives

Descriptive analyses (see Table A2 of the SI Appendix) show that the mean psychological distress score (sample mean = 3.14 [95% CI 3.05–3.22], population mean = 3.37 [3.24–3.51]) is slightly above the threshold for the PHQ-4’s cutoff for mild distress and well above the average of 1.76 (95% CI 1.7–1.81) previously established for the general German population [27]. Mean life satisfaction is 7.26 (7.19–7.33) in our sample and 6.9 (6.78–7.02) in the population—similar to means found in the German general population (e.g. mean = 6.98 [SD = 0.78], [48]). Tables A2 and A3 also shows descriptive statistics for all independent variables in the following analyses.

### Main Analyses

#### Sociodemographic and Pre-/Peri-migration Control Variables

Figure 1 shows that several sociodemographic and pre-/peri-migration stressors relate to psychological distress and life satisfaction. Being female, older, Afghan or of an 'Other' nationality, being single, divorced or widowed are associated with increased psychological distress across Models 1–3a and 1–3b. Being Eritrean is associated with decreased psychological distress across models. A longer time in Germany is associated with decreased distress and increased life satisfaction in Models 1a and 2a. Being male, younger, more educated, Christian, single or divorced/widowed are all associated with decreased life satisfaction in Models 1–3b, whereas being Afghan and Eritrean appears to correlate with greater satisfaction. In the category of pre- and peri-migration stressors, those reporting a greater number of flight reasons and having had adverse experiences during flight exhibit elevated distress and reduced life satisfaction across models. The addition of these pre-/peri-migration factors constitutes a significant, albeit small improvement in model fit.

#### Integration Measures and Post-migration Living Conditions

Adding post-migration contextual factors again constitutes a significant improvement in model fit, with a greater increase in R^2^ in the life satisfaction than in the distress model. The legal outcomes “protection” and “suspension of deportation”, both of which grant a mere one-year right to stay, are linked to elevated levels of psychological distress compared to the positive outcome of being granted the status of refugee or asylee. However, neither is linked to life satisfaction. Crucially, awaiting the outcome of the legal proceedings, either for the initial asylum application or after an appeal against a negative decision, is associated with significantly higher levels of psychological distress and lower life satisfaction compared to the positive response of having a refugee or asylum status. Those seeking to reunite with underage children or with a spouse living outside Germany are more distressed and less satisfied with life than those not seeking family reunification.

Housing conditions are significantly associated with our outcome measures. Private housing is related to lower levels of psychological distress and higher levels of life satisfaction compared to residence in refugee housing facilities. Furthermore, being in the workforce is associated with reduced levels of distress. Interestingly, however, employment does not relate to life satisfaction according to our analysis. Finally, more time spent with the native German population and better German language skills are associated with lower levels of distress and increased life satisfaction.

#### Exploration of Interaction Effects

As shown in Table 1, in our complete model for psychological distress, interactions between gender and seeking family reunification, employment status, course participation, time with co-nationals, and German language are significant at α = 0.05. Stratification by gender revealed that seeking family reunification is only significantly associated with elevated distress in males, but still trending for females. Only employed male respondents experience lower levels of distress. Regarding participation in integration courses, associations with distress have opposite though insignificant effects for females and males. Females who spend more time with co-nationals experience reduced distress, unlike male. Finally, higher German language ability is related more strongly to reduced distress in male. For life satisfaction, gender interacts with family reunification and being in education, with a significant negative association between family reunification and life satisfaction in male, but not in female respondents, and, conversely, a negative association between currently being in education in females but not in males. Finally, time spent with co-nationals has an opposite relationship to life satisfaction for males and females. We found no significant interactions with age.

**Table 1:** Interactions between gender, age, nationality, and level of education and the post-migration variables that were significant at α = 0.05

	Psychological distress	Life satisfaction
**Interactions with gender**		
*× Seeking family reunification*	Wald = 4, p = 0.046	Wald = 5.286, p = 0.022
Stratified female (seeking)	Beta = 0.542, CI − 0.013; 1.096	Beta = − 0.357, CI − 0.773; 0.078
Stratified male (seeking)	Beta = 1.276, CI 0.902; 1.651	Beta = 1.128, CI − 1.459; − 0.797
*× Currently in education*		Wald = 3.951, p = 0.047
Stratified female (in education)		Beta = − 0.715, CI − 1.326;-0.105
Stratified male (in education)		Beta = 0.123, CI − 0.246; 0.493
*× Employment*	Wald = 5.1, p = 0.006	
Stratified female (employed)	Beta = 0.274, CI − 0.432; 0.98	
Stratified male (employed)	Beta = − 0.513, CI − 0.83; − 0.2	
*× Course participation*	Wald = 4.32, p = 0.038	
Stratified female (course participation)	Beta = 0.058, CI − 0.109; 0.225	
Stratified male (course participation)	Beta = − 0.542, CI − 0.164; 0.056	
*× Time with co-nationals*	Wald = 5.22, p = 0.02	Wald = 3.841, p = 0.05
Stratified female (amount of time)	Beta = − 0.236, CI − 0.384; − 0.09	Beta = 0.075, CI − 0.036; 0.186
Stratified male (amount of time)	Beta = − 0.054, CI − 0.164; 0.058	Beta = − 0.072, CI − 0.174; 0.03
*× German language ability*	Wald = 4.33, p = 0.037	
Stratified female (German language ability)	Beta = − 0.107, CI − 0.286; 0.072	
Stratified male (German language ability)	Beta = -0.236, CI − 0.384;-0.09	
**Interactions with nationality**		
*× Legal status*	Wald = 3.067, p = 0.016	
Stratified Syrian (insecure/waiting)	Beta = 0.288, CI 0.014; 0.563	
Stratified Afghan (insecure/waiting)	Beta = 0.746, CI 0.137; 1.355	
Stratified Iraqi (insecure/waiting)	Beta = 1.017, CI 0.446; 1.587	
*× Type of accommodation*	Wald = 3.294, p = 0.01	Wald = 3.091, p = 0.015
Stratified Syrian (private accommodation)	Beta = − 0.521, CI − 0.841; − 0.2	Beta = 0.759, CI 0.487; 1.03
Stratified Eritrean (private accommodation)	Beta = 0.763, CI 0.043; 1.484	Beta = − 0.419, CI − 1.142; 0.304
*× Employment*	Wald = 2.158, p = 0.027	
Stratified Syrian (employed)	Beta = − 0.178, CI − 0.581; 0.225	
Stratified Other (employed)	Beta = − 0.883, CI − 1.483; − 0.282	
*× Course participation*	Wald = 2.42, p = 0.046	
Stratified Syrian (course participation)	Beta = 0.068, CI − 0.057; 0.193	
Stratified Other (course participation)	Beta = − 0.088, CI − 0.304; 0.128	
*× Time with co-nationals*	Wald = 3.362, p = 0.009	
Stratified Syrian (amount of time)	Beta = − 0.06, CI − 0.187; 0.068	
Stratified Other (amount of time)	Beta = − 0.369*, CI − 0.588; − 0.15	
*× German language ability*		Wald = 3.399, p = 0.009
Stratified Syrian (German language ability)		Beta = 0.224, CI 0.103; 0.345
Stratified Afghan (German language ability)		Beta = − 0.121, CI − 0.374; 0.131
**Interactions with level of education**		
*× Currently in education*	Wald = 2.644, p = 0.071	Wald = 3.453, p = 0.032
Stratified low level (in education)	Beta = 0.352, CI − 0.175; 0.879	Beta = 0.245, CI − 0.197; 0.0687
Stratified high level (in education)	Beta = 0.98, CI 0.116; 1.845	Beta = − 0.66, CI − 1.346; 0.026

Wald test results comparing linear regression models predicting psychological distress or life satisfaction (complete regression models, including sociodemographic and pre-/peri-migration controls and all post-migration factors, as in Models 3a and 3b, pooled from 10 imputed datasets) with and without each interaction term (each term added to the model on its own) as well as regression terms and 95% confidence intervals (CI) for follow-up stratifications

Nationality interacts with several post-migration factors in its association with psychological distress. Afghans and Iraqis with insecure legal statuses experience greater increases in distress compared to Syrians with this status. Unlike other nationalities, Eritreans who live in private accommodation actually experience greater levels of distress and lower life satisfaction than fellow nationals living in refugee housing facilities. ‘Other’ nationalities exhibit slightly different patterns of associations, with employment status and time spent with co-nationals being significantly associated with lower levels of distress only in this group. Participation in integration courses is related to distress in the opposite direction for ‘Others’ compared to Syrians. German language ability is only significantly related to higher life satisfaction among Syrians.

Education interacts with currently being in education in predicting life satisfaction. Highly educated respondents who are in education are less satisfied; there is no such relationship in respondents with low or medium levels of education.

We also found several interactions with the number of flight reasons (Table 2) (our indicator of traumatic experiences). The more flight reasons respondents report, the stronger the relationship between seeking family reunification and living in refugee housing facilities and elevated distress, as well as living in refugee housing facilities and reduced life satisfaction. Also, the higher the number of flight reasons, the more distressing currently being in education appears to be and the more distress-reducing spending time with Germans. Finally, having multiple reasons for flight is associated with an increase in the positive association between language and life satisfaction. It should be noted that none of our interaction effects would be statistically significant under standard corrections for multiple comparisons.

**Table 2:** Interactions between number of flight reasons and the post-migration variables that were significant at α = 0.05

	Psychological distress	Life satisfaction
**Interactions with number of flight reasons**		
*× Seeking family reunification*	Wald = 4.905, p = 0.027	
Stratified one or none (seeking)	Beta = 0.82, CI 0.426; 1.213	
Stratified two or three (seeking)	Beta = 1.316, CI 0.695; 1.936	
Stratified four or five (seeking)	Beta = 1.276, CI 0.902; 1.651	
* × Type of accommodation*	Wald = 4.852, p = 0.028	Wald = 4.036, p = 0.045
Stratified one or none (private accom.)	Beta = − 0.299, CI − 0.577; − 0.02	Beta = 0.455, CI 0.228; 0.682
Stratified two or three (private accom.)	Beta = − 0.586, CI − 1.019; − 0.152	Beta = 0.271, CI − 0.094; 0.636
Stratified four or five (private accom.)	Beta = − 0.660, CI − 1.010; − 0.310	Beta = 0.812, CI 0.534; 1.090
*× Currently in education*	Wald = 5.651, p = 0.017	
Stratified one or none (in education)	Beta = − 0.053, CI − 0.525; 0.418	
Stratified two or three (in education)	Beta = 0.535, CI − 0.196; 1.267	
Stratified four or five (in education)	Beta = 0.901, CI 0.284; 1.519	
*× Time with Germans*	Wald = 5.268, p = 0.022	
Stratified one or none (time with Germans)	Beta = − 0.112, CI − 0.234; 0.009	
Stratified two or three (time with Germans)	Beta = − 0.209, CI − 0.398; − 0.020	
Stratified four or five (time with Germans)	Beta = − 0.283, CI − 0.434; − 0.132	
*× German language ability*		Wald = 4.393, p = 0.036
Stratified one or none (German language ability)		Beta = 0.078, CI − 0.036; 0.191
Stratified two or three (German language ability)		Beta = 0.131, CI –0.051; 0.313
Stratified four or five (German language ability)		Beta = 0.186, CI 0.047; 0.326

Wald test results comparing linear regression models predicting psychological distress or life satisfaction (complete regression models, including sociodemographic and pre-/peri-migration controls and all post-migration factors, as in Models 3a and 3b, pooled from 10 imputed datasets) with and without each interaction term (each term added to the model on its own) as well as regression terms and 95% confidence intervals (CI) for follow-up stratifications, for which the numeric variable was split into three categories: no or one evidently potentially distressing flight reason, two or three, four or five

## Discussion

Overall, our results support and specify previous claims linking refugees’ mental health and well-being in the first years after arrival to post-migration living conditions, many of which are subject to integration policies. In particular, our study shows that after controlling for key sociodemographics as well as pre- and peri-migration stressors, the legal hurdles refugees face while securing their future life in the host country are related to higher levels of psychological distress. Policy makers should thus consider the potentially negative impact of an uncertain legal status, acknowledging that a large proportion of refugees who are granted a less secure status (mostly cases of subsidiary protection) end up having this status renewed, still remaining in their host country for several years [49]. This is further corroborated by our finding that refugees who are awaiting the outcome of the asylum process exhibit both higher levels of distress and lower life satisfaction compared to those with a relatively secure legal status. This is consistent with previous studies indicating the detrimental consequences of lengthy asylum procedures for mental health [50]. The much criticized [51, 52] lack of full access to healthcare for asylum seekers in many countries becomes even more problematic in light of these findings. Our results suggest that policies facilitating family reunification could enhance life satisfaction and reduce psychological distress among refugees. While the UN Refugee Convention states that family unity is among the essential rights of refugees, and Article 8 of the European Convention on Human Rights calls for flexible and prompt decision making, many European countries have restricted the options for reunification since 2015 [53]. Mental health care professionals working with refugees should be briefed on their patients’ possible legal battles.

Looking beyond these legal aspects, we find that living in refugee rather than in private accommodation is associated with greater distress and reduced life satisfaction. Although self-selection might play an important role here, it seems plausible that residing in refugee housing facilities, which often means living in crowded quarters with limited privacy, restricted autonomy, and isolation from the local community, in fact causes or exacerbates health issues, as has been previously examined in detail [54]. Residing in refugee housing facilities may also come with safety concerns, for example in light of the frequency of attacks on refugee accommodation in many host countries [55]. Beyond efforts to improve living conditions in refugee housing facilities, the strengthening of infrastructural links between these facilities and psychosocial services would be an adequate response to this finding.

Whilst being employed is associated with reduced psychological distress in our study, as well as in other studies [56], it is not linked to higher levels of satisfaction as in most studies using general population samples [57]. These cases in which measures related to mental health and well-being diverge demonstrate that many of the established integration measures miss the emotional toll of certain circumstances [58]. The lack of a link between employment and life satisfaction here might be due to the expectations of refugees regarding the norm of being employed. In contrast to the native population, in which being part of the workforce is the social norm, refugees might have different expectations, particularly in the first years after arrival. The association between unemployment and distress applies to other populations as well [59]. It is thought to be a bidirectional relationship, calling for a similar reciprocal relationship in employment and health policies [60], especially in the case of a vulnerable population like the one at hand.

Finally, like some previous studies [61], our study shows that contact with the native population and host country language ability are associated with distress and life satisfaction. As with employment, the causal direction of this relationship is just as likely one or the other. It is noteworthy that time spent with Germans is positively associated with both distress and life satisfaction, while time spent with non-relatives from the country of origin and with people from other countries is related to neither. This suggests that it is interactions with the host population, specifically, that relate to distress and life satisfaction. The relationship between German language ability and our outcome measures underscore the importance of addressing the language barrier in refugees’ access to mental health services [62]. If those with lower levels of German language ability are more distressed, the language barrier is an even more pressing issue.

Our analyses also show that several sociodemographic as well as pre- and peri-migration stressors moderate associations between post-migration living conditions and psychological distress and life satisfaction. Our exploratory analyses suggest that this is the case for gender, nationality, level of education, and the number of flight reasons. Future research should address these potential moderations.

### Limitations

The primary limitation of our study is the correlative nature of the evidence. Our study design did not allow for conclusions about a causal relationship between living conditions and mental health and well-being. We have also limited our study to examining post-migration living conditions captured by the survey data we used that are amenable to host country integration policy measures. There are, of course, many descriptors of life in the host country beyond these factors, also including the cultural dimensions of the acculturation process. Furthermore, whilst the cross-cultural validity of the PHQ-4 has been tested in Arabic-speaking refugees in Germany [27], the validity of mental health scales across cultural backgrounds is contentious [63, 64]. Given the size of the survey, outcome measures need to be brief. While both of our measures have shown good reliability and validity, the brevity of our scales is a limitation. In the case of psychological distress, for example, the four-item screener only measures the central symptoms of depression and anxiety, not other symptoms such as somatization. A selection bias favoring those with higher levels of mental health and well-being is also likely to underlie sampling for this survey [65]. Finally, the applicability of our findings to other host societies is questionable, considering the vast differences in policies and other contingencies even between Western European countries. Nonetheless, Germany is a highly relevant case because it has adopted the largest number of refugees in the European Union. By the end of 2016, the population of refugees reached 1.3 million people, with 441,900 new asylum applications submitted in 2015 and 722,400 claims made in 2016 [66].

### New Contributions to the Literature

In summary, our study finds that greater certainty and stability, in the form of a secure legal status, non-temporary housing, family reunification, and social anchoring in the host society through language abilities and contacts are linked to certain aspects of better mental health and well-being in the early years after arrival. To our knowledge, these associations have not been shown in a similarly large, rigorously collected survey dataset on newly arrived refugees.

## Electronic supplementary material

Below is the link to the electronic supplementary material.

Supplementary material 1 (PDF 1154 kb)
