# Supplementary material for: Living Conditions and the Mental Health and Well-being of Refugees: Evidence from a Large-Scale German Survey
Source: J Immigr Minor Health. 2020 Jan 24;22(5):903–13. doi: 10.1007/s10903-019-00968-5 (PMC7441051; doi:10.1007/s10903-019-00968-5)
Supplement: Supplementary file 1 — Supplementary material 1 (PDF 1154 kb) [file 10903_2019_968_MOESM1_ESM.pdf]

Supplementary Information Appendix for
Living conditions and the mental health and well-being of refugees: Evidence from a large-scale German survey

This SI Appendix includes:
Data access details
Variable composition details
Interaction Analysis Details
Tables A1 to A8

Data Access

All data used in this study is provided to the international research community via the SOEP Research Data Center (RDC) at the German Institute for Economic Research (DIW Berlin), Germany for scientific purposes upon signing a data distribution contract.

Variable composition details

In this section, we break down exactly how all of the variables that were used in this study were coded, the IAB-BAMF-SOEP questionnaire wording of the items underlying variables where relevant, as well as the IAB-BAMF-SOEP-given variable name ('bgprXZY' for questions from the Individual and Biography Questionnaire; 'bghrXYZ' for questions from the Household Questionnaire).

Outcome Variables
Psychological Distress. Measured as the total score of the four-item PHQ-4 scale. The wording of the overall scale question in the IAB-BAMF-SOEP questionnaire reads "Now let's talk about the last two weeks. How often have you felt negatively affected by the following complaints in the last two weeks?" Wording of scale item #1: "Little interest or pleasure in your activities" (bgpr312, part of two-item depression subscale); wording of scale item #2: "Low spirits, melancholy or hopelessness?" (bgpr313, part of two-item depression subscale); wording of scale item #3: "Nervousness, anxiety or tension?" (bgpr314, part of two-item anxiety subscale); wording of scale item #4: "Unable to stop or control worrying?" (bgpr315, part of two-item anxiety subscale). We coded the responses to these items as numeric values between 0 ('not at all') and 3 ('(almost) every day') or missing values. We coded the composite distress variable as missing if one or more of the four constituting items were missing.
Life Satisfaction. Measured as the score of a single-item global life satisfaction scale. The wording of the scale in the IAB-BAMF-SOEP questionnaire reads "How satisfied are you currently with your life in general?" (bgpr457). We coded the responses to this scale as numeric values between 0 ('totally dissatisfied') and 10 ('totally satisfied') or missing values.

Independent Variables
Federal state of residence. This variable (nuts116) is taken from the generated household variables provided by the SOEP.
Gender. Measured as a binary categorical variable. The wording of the scale in the IAB-BAMF-SOEP questionnaire reads "Your gender" (bgpr_l_0101). We coded the responses to this item as factor levels 'female', 'male' or missing values.

Age. Measured in years as the birth year (bgpr_l_0103) subtracted from the year of the survey interview (2016). Implausible values were defined as missing values.
Level of education. Measured using the composite variable created by the SOEP (isced11_16) on the basis of the International Standard Classification of Education of 2011. We combined the categories 'in school', 'primary education' and 'lower secondary education' into the category 'low'; the categories 'upper secondary education', 'post-secondary non-tertiary education' and 'short-cycle tertiary education' into the category 'medium' and the categories 'Bachelors or equivalent level', 'masters or equivalent level' and 'doctoral or equivalent level' into the category 'high'.
Time in Germany. Measured in years as the year of arrival (bgpr_l_3401) plus the month of arrival (bgpr_l_3402) represented as a numeric value between 1 and 12 divided by 12 subtracted from the year of the survey interview (2016) plus the month of the interview (bgprmonin) represented as a numeric value between 1 and 12 divided by 12. The time in Germany variable was coded as missing in cases where either the item measuring the year of arrival or the item measuring the month of arrival was a missing value. There were no missing values in the variable measuring the month of the interview.
Religious affiliation. Variable bgpr350, which asks about religious affiliation.
Marital status. Variable bgpr390. We combined the categories 'divorced' and 'widowed'.
Flight reasons. Based on variables bgpr_l_3601-bgpr_l_3611.
Arrived in Germany alone. Variable bgpr_l_3501.
Negative flight experiences. Based on variables bgpr_l_3301-bgpr_l_3308.
Legal status. The categorical legal status variable was created based on the IAB-BAMF-SOEP survey question reading "Has your application for asylum been approved by the Federal Office for Migration and Refugees?" (bgpr47). We combined the first two categories ("Yes, I have been assigned refugee status" and "Yes, my entitlement to asylum has been recognized") into 'refugee or asylum status'. We added another category for those respondents who were still awaiting an outcome from the application process – whose response to the question "Has an official decision regarding your application for asylum been made yet by the Federal Office for Migration and Refugees?" (bgpr45) was "no". We excluded all 76 respondents who reported "No, my application was rejected and I was asked to leave Germany", except those 49 who reported that they were still awaiting the outcome of their appeal to the decision of the Federal Office (Q: "Was your court action against the German Federal Office for Migration and Refugees successful", A: "No, a decision is yet to be made regarding my court action", bgpr49). These respondents were also included in the 'awaiting outcome' category. We furthermore added those 141 respondents who reported that they did not go through the asylum procedure ("This does not apply to me because I did not go through an asylum procedure (e.g. resettlement or entry on humanitarian grounds" in response to "When were your personal de- tails recorded and when were you issued with an asylum seeker registration certificate (BÜMA) or proof of arrival?" (bgpr3903)) to the safest status category, 'refugee or asylum status'. We assigned missing values to those 99 individuals who did not respond to bgpr45, i.e. whether an official decision regarding their application for asylum had been made, who subsequently ended up in the 'not applicable' category in bgpr47.
Family reunification. The categorical family reunification variable was created by ascertaining whether any of the eight eldest children of respondents that the IAB-BAMF-SOEP questionnaire asks about were born after the year 1998 ("In what year was your nth child born?", bgpr_l_403, 407, 411, 415, 419, 423, 427, 431) and whether the respondent is planning to bring at least one of these children born after 1998 to Germany ("Are you planning to bring this child to Germany?", bgpr_l_406, 410, 414, 418, 422, 426, 430, 434) or whether the respondent is planning to bring his/her spouse/registered partner to Germany ("Are you planning to bring you spouse/registered partner to Germany?", bgpr_l_392). The resulting binary family reunification variable assigns a 'yes'-value

to reports of having either a spouse or any number of children born after 1998 whose relocation to Germany is planned, 'no'-values to reports of having neither and missing values for all respondents with missing responses in either any of the constituent variables on reunification with children or any of the constituent variables on reunification with a spouse/registered life partner. Our choice to include only spouses or registered partners and minor children was based on German family reunification law, which only potentially allows for family reunification with these parties (section 26 of the Asylum Act (*AsylG*) (1), Section 36 a of the Residence Act (2)).

Type of accommodation. Determined by bringing together two questions from the household questionnaire, the one reading "In what type of accommodation does the interviewee live?" (bghr01), the other reading "Are there further apartments in this building in which refugees live?" (bghr02). We created a new composite variable with one category for those respondents who reported living in shared accommodation, another category for those respondents who reported living in private accommodation (private apartment or private house) with one or more other apartments inhabited by refugees in their building and a third category for those respondents who reported living in private accommodation without other refugees in their building.

Currently in education. Currently in education is a binary categorical variable based on answers to the survey question reading "Are you currently in training? This means: Are you attending a school or college / university (including doctoral studies), are you taking vocational training or are you taking a continuing professional development course?" (bgpr292).

Currently working. Currently working is a three-level categorical variable that we created based on answers to the survey questions reading "Are you currently working?" (bgpr161) and "Are you planning to work (again) in the future?" (bgpr162). We counted anyone reporting working in any capacity (including full-time and part-time employment, minimal or irregular employment, in-company training / apprenticeship or occupational retraining, internships) as working and the rest as not working. We then added a third level, putting those who currently are not working and reported that it's unlikely or ruled out that they will work in the future in their category, resulting in the categories 'no', 'yes', 'likely or certainly will not work'.

Course participation. Course participation is a numeric variable that we created counting the total number of language and integration courses respondents reported having attended. The courses asked about were an integration course organized by the German Federal Ministry for Migration and Refugees (BAMF) (bgpr94), an ESF BAMF course to learn vocational German (bgpr100), an entry course for German language skills organized by the German Federal Employment Agency (bgpr106), the "Perspectives for Refugees" course organized by the German Federal Employment Agency (bgpr112), the "Perspectives for Young Refugees" course organized by the German Federal Employment Agency (bgpr118) and any other German language courses (bgpr124). We assigned the numeric value '1' to reports of having attending each course, '0' to reports of not having attending, and missing values. We then summed these six numeric variables. Only those respondents who had missing values on all six courses were assigned missing values in the composite variable.

Time with people from respondents' country of origin, Germans and people from another country. The three variables on amount of time spend with three different groups are numeric variables on a scale from 1 ('never') to 6 ('every day') that represent responses to the questions "How often do you spend time with people from your country of origin who are not related to you?" (bgpr210), "How often do you spend time with German people?" (bgpr211) and "How often do you spend time with people from other countries?" (bgpr212). German language ability. Measured as the mean scores of the 1 ('not at all') through 5 ('very well') responses to the questions "How well can you speak German?" (bgpr87), "How well can you write in German?" (bgpr88) and "How well

can you read in German?" If any of these three responses are missing, the composite variable 'German language ability' is also a missing value.

Auxiliary Variables

Responses to the following questions were added into our imputation model as auxiliary variables with only minor transformations (for example, from a categorical into a numeric variable, missing value category added):

Questions on country of origin background
•"And how strongly do you feel connected with your country of origin?" (bgpr325)
Questions related to level of education
•"How well can you write in your native language?" (bgpr_l_74)
•"How well can you read in your native language?" (bgpr_l_75)
•"Were you in vocational training in a country other than Germany or did you study (at higher education level) in a country other than Germany?" (bgpr_l_231)
Questions on flight
•"Did any relatives or acquaintances who already lived in Germany help you move to Germany?" (bgpr_l_3801-3)
•"When were your personal details recorded and when were you issued with an asylum seeker registration certificate (BÜMA) or proof of arrival?" (bgpr3901)
Questions on level of awareness of German services and institutions
•"Do you know about the refugee and asylum advice services available?" (bgpr140)
•"Do you know the Migration Advice Service for Adult Immigrants (MBE)?" (bgpr141)
•"Do you know the Youth Migration Services (JMD)?" (bgpr142)
•"Do you know the general employment market advisory service at the German Federal Employment Agency [Bundesagentur für Arbeit], the Jobcenter?" (bgpr143)
•"Do you know about the job advisory service at the German Federal Employment Agency [Bundesagentur für Arbeit], the Jobcenter?" (bgpr144)
Questions related to experience of Germany
•"How often have you personally experienced being disadvantaged in Germany because of your origin?" (bgpr67)
•"Would you like to stay in Germany permanently?" (bgpr68)
•"And how is it now: Do you feel welcome in Germany now?" (bgpr327)
Questions related to health (mostly from the 12-Item Short Form Health Questionnaire)
•"How satisfied are you with your current health?" (bgpr298)
•"How would you describe your current state of health?" (bgpr299)
•"And what about other strenuous activities in everyday life, e.g. when you have to lift something heavy or need to be mobile: Does your state of health restrict you a lot, a little or not at all?" (bgpr301)
•"How often in the last four weeks did you feel rushed or under time pressure?" (bgpr302)
•"How often in the last four weeks did you feel in low spirits and melancholy?" (bgpr303)
•"How often in the last four weeks did you feel calm and balanced?" (bgpr304)
•"How often in the last four weeks did you feel full of energy?" (bgpr305)
•"How often in the last four weeks did you suffer from severe physical pain?" (bgpr306)
•"How often in the last four weeks, due to health problems of a physical nature, did you achieve less in your work or everyday activities than you actually intended?" (bgpr307)

•"How often in the last four weeks, due to health problems of a physical nature, have you been restricted in the type of tasks you can perform in your work or everyday activities?" (bgpr308)•
"How often in the last four weeks, due to psychological or emotional problems, did you achieve less in your work or everyday activities than you actually intended?" (bgpr309)
• "How often in the last four weeks, due to psychological or emotional problems, did you perform your work or everyday activities less carefully than usual?" (bgpr310)
•"How often in the last four weeks, due to health or psychological problems, have you been restricted in terms of your social contact to for example friends, acquaintances or relatives?" (bgpr311)
•Resilience. Measured as the total score of the four-item Brief Resilience Scale (bgpr345, bgpr346, bgpr347, bgpr347).

Questions related to personality and attitude toward self
•"I have a positive attitude about myself." (bgpr344)
•"How do your rate yourself personally? In general, are you someone who is ready to take risks or do you try to avoid risks?" (bgpr349)
Questions related to feelings of social isolation
•"How often do you feel that you miss the company of others?" (bgpr321)
•"How often do you feel like an outsider?" (bgpr322)
•"How often do you feel socially isolated?" (bgpr323)
•"How often do you feel that you miss people from your country of origin?" (bgpr324)
•"Are you worried that you will be unable to stay in Germany?" (bgpr358)
•"Are you worried that you will be unable to return to your country of origin?" (bgpr359)

Interaction analysis details

Before introducing interaction terms into our models, we cross-tabulated our to-be-interacted categorical variables, setting a minimum cell size of n=30 and making necessary adjustments to variable specifications (e.g. employed "yes" or "no" rather than "yes", "no", "not seeking employment" in some cases). We added all interaction terms to our complete models one at a time and assessed their effect using Wald tests comparing the model with and without each interaction term. To examine significant interactions further, we stratified our regressions. In order to stratify, we transformed some of our numeric variables into categorical ones (number of flight reasons).

References

1. Germany: Asylum Act (AsylVfG) 2008 [Germany], 2 September 2008, available at: https://www.refworld.org/docid/48e4e9e82.html (accessed 2 September 2019)
2. Germany: Residence Act of 2008 [Germany], 25 February 2008, available at: https://www.refworld.org/docid/48e5cd7f2.html (accessed 2 September 2019)

Tables A1-A8

	Percent missing
Distress	9.39
Life satisfaction	0.97
Federal state of residence	0.00
Gender	0.00
Age	0.05
Education level	7.54
Nationality	0.00
Religious affiliation	2.45
Marital status	0.76
Time since arrival	3.65
Number of flight reasons	1.32
Arrived in Germany alone	0.16
Negative flight experiences	0.81
Legal status	0.93
Family reunification plans	1.41
Accommodation	1.91
Currently in education	0.51
Currently working	0.00
Course participation	0.23
Time with co-nationals	1.09
Time with Germans	0.79
Time with nationals	1.04
German language skills	0.09

Table A1. Percentage of values missing per variable for all dependent and independent variables.

Variable Name (Range)	Unweighted Means (SD) /Proportions	Unweighted 95%-CI	Weighted Means (SD) /Proportions	Weighted 95%-CI
Dependent Variables				
Psychological Distress (0-12)	3.136 (2.787)	3.051-3.221	3.374 (2.878)	3.235-3.513
Life Satisfaction (0-10)	7.259 (2.334)	7.189-7.329	6.896 (2.457)	6.778-7.015
Sociodemographic Controls				
Male (0/1)	0.621	0.607-0.636	0.735	0.717-0.754
Female (0/1)	0.379	0.364-0.393	0.265	0.246-0.283
Age (18-76)	33.57 (10.39)	33.26-33.879	30.944 (10.467)	30.453-31.436
Low level of education (0/1)	0.614	0.6-0.629	0.587	0.563-0.611
Medium level of education (0/1)	0.212	0.199-0.225	0.24	0.217-0.263
High level of education (0/1)	0.174	0.162-0.185	0.173	0.156-0.19
Syrian 0/1	0.499	0.484-0.514	0.412	0.39-0.434
Afghan (0/1)	0.128	0.118-0.138	0.139	0.122-0.157
Iraqi (0/1)	0.124	0.114-0.134	0.088	0.078-0.097
Eritrea (0/1)	0.055	0.049-0.062	0.048	0.041-0.056
Other (0/1)	0.193	0.182-0.205	0.312	0.287-0.337
Muslim (0/1)	0.713	0.699-0.727	0.71	0.688-0.732
Christian (0/1)	0.149	0.138-0.16	0.166	0.147-0.184
Other religious affiliation (0/1)	0.074	0.066-0.081	0.052	0.043-0.062
No religious affiliation (0/1)	0.064	0.057-0.072	0.072	0.06-0.084
Married (0/1)	0.661	0.647-0.675	0.455	0.432-0.479
Single (0/1)	0.295	0.281-0.309	0.502	0.478-0.526
Divorced/Widowed (0/1)	0.044	0.038-0.05	0.043	0.033-0.052
Time in Germany (0 - 3.92 years)	1.582 (0.77)	1.559-1.605	1.35 (0.709)	1.317-1.383
Pre-/Peri-migration Controls				
Number of flight reasons (0-5)	2.357 (1.388)	2.315-2.398	2.27 (1.406)	2.202-2.337
Arrived in Germany alone - no (0/1)	0.755	0.742-0.768	0.581	0.556-0.605
Arrived in Germany alone - yes (0/1)	0.245	0.232-0.258	0.419	0.395-0.444
No negative flight experiences (0/1)	0.337	0.322-0.351	0.3	0.279-0.321
Negative flight experiences (0/1)	0.34	0.326-0.354	0.365	0.341-0.388
No answers on flight experience (0/1)	0.323	0.309-0.337	0.335	0.313-0.358
Integration Measures and Post-migration Living Conditions				
Asylum/Refugee status (0/1)	0.552	0.537-0.566	0.454	0.431-0.477
Subsidiary protection (0/1)	0.031	0.026-0.037	0.024	0.018-0.03
Suspension of deportation (0/1)	0.02	0.016-0.024	0.019	0.012-0.026
Awaiting outcome (0/1)	0.397	0.382-0.411	0.503	0.479-0.527
Not seeking family reunification (0/1)	0.909	0.9-0.918	0.875	0.859-0.891
Seeking family reunification (0/1)	0.091	0.082-0.1	0.125	0.109-0.141
Refugee housing facility (0/1)	0.339	0.325-0.353	0.475	0.451-0.5
Private accommodation (0/1)	0.661	0.647-0.675	0.525	0.5-0.549
Not currently in education (0/1)	0.945	0.938-0.952	0.939	0.928-0.95
Currently in education (0/1)	0.055	0.048-0.062	0.061	0.05-0.072
Currently not working (0/1)	0.82	0.809-0.832	0.823	0.804-0.841
Currently working (0/1)	0.104	0.095-0.113	0.119	0.104-0.135
Not seeking work (0/1)	0.076	0.068-0.084	0.058	0.047-0.069
Number of courses attended (0-5)	0.851 (0.685)	0.831-0.871	0.848 (0.698)	0.816-0.881
Time with co-nationals (1-6)	3.868 (1.839)	3.813-3.923	4.168 (1.851)	4.08-4.255
Time with Germans (1-6)	3.679 (1.869)	3.623-3.735	3.699 (1.898)	3.61-3.789
Time with others (1-6)	2.88 (1.926)	2.822-2.937	3.23 (1.967)	3.137-3.322
German language ability (1-5)	2.581 (0.967)	2.552-2.61	2.594 (0.966)	2.55-2.637

Table A2. Descriptive statistics for dependent variables, sociodemographic control variables, pre- and peri-migration control variables, and integration measures and post-migration living conditions. SD = standard deviation, CI = confidence interval. Descriptives for the control variable federal state of residence can be found in Table A2 of the SI Appendix. All values come from imputed data.

	Unweighted Proportions	Unweighted 95%-CI	Weighted Proportions	Weighted 95%-CI
Baden-Württemberg	0.14	0.129-0.15	0.12	0.106-0.134
Bavaria	0.146	0.135-0.156	0.126	0.112-0.139
Berlin	0.068	0.06-0.075	0.042	0.035-0.05
Brandenburg	0.05	0.044-0.057	0.031	0.024-0.037
Bremen	0.013	0.009-0.016	0.025	0.013-0.036
Hamburg	0.022	0.018-0.027	0.03	0.021-0.039
Hesse	0.073	0.065-0.081	0.077	0.065-0.088
Meckelenburg-Vorpommern	0.02	0.016-0.024	0.016	0.011-0.021
Lower Saxony	0.086	0.077-0.094	0.103	0.088-0.118
North Rhine-Westphalia	0.179	0.167-0.19	0.242	0.219-0.265
Rhineland-Palatinate	0.033	0.027-0.038	0.026	0.02-0.032
Saarland	0.033	0.028-0.039	0.04	0.031-0.048
Saxony	0.041	0.035-0.047	0.033	0.026-0.04
Saxony-Anhalt	0.023	0.019-0.028	0.024	0.017-0.03
Schleswig-Holzstein	0.052	0.045-0.058	0.046	0.037-0.055
Thuringia	0.022	0.018-0.027	0.022	0.016-0.028

Table A3. Descriptive statistics for federal state of residence. All values come from imputed data.

	Dependent variable:		
	Psychological Distress		
	Model 1a	Model 2a	Model 3a
	Beta Coef. (95%-CI)	Beta Coef. (95%-CI)	Beta Coef. (95%-CI)
Intercept	2.946*** (2.679, 3.212)	2.513*** (2.225, 2.801)	2.509*** (2.171, 2.846)
Bavaria (1)	−0.465*** (−0.776, −0.153)	−0.443*** (−0.755, −0.130)	−0.377** (−0.688, −0.066)
Berlin (1)	−0.130 (−0.518, 0.259)	−0.098 (−0.484, 0.289)	−0.251 (−0.635, 0.133)
Brandenburg (1)	−0.176 (−0.606, 0.255)	−0.156 (−0.583, 0.271)	−0.142 (−0.564, 0.279)
Bremen (1)	−0.680* (−1.445, 0.085)	−0.548 (−1.309, 0.213)	−0.360 (−1.116, 0.397)
Hamburg (1)	−0.797*** (−1.396, −0.197)	−0.679** (−1.274, −0.084)	−0.717** (−1.305, −0.129)
Hesse (1)	−0.649*** (−1.032, −0.265)	−0.570*** (−0.955, −0.184)	−0.500** (−0.881, −0.120)
Meckelenburg-Vorpommern (1)	−0.057 (−0.690, 0.575)	0.056 (−0.573, 0.685)	0.190 (−0.431, 0.811)
Lower Saxony (1)	−0.462** (−0.826, −0.097)	−0.497*** (−0.858, −0.135)	−0.263 (−0.625, 0.099)
North Rhine-Westphalia (1)	−0.434*** (−0.728, −0.140)	−0.342** (−0.639, −0.045)	−0.207 (−0.504, 0.091)
Rhineland-Palatinate (1)	0.647** (0.142, 1.151)	0.604** (0.103, 1.105)	0.769*** (0.271, 1.266)
Saarland (1)	−0.280 (−0.789, 0.230)	−0.292 (−0.799, 0.214)	−0.032 (−0.539, 0.474)
Saxony (1)	−0.306 (−0.774, 0.163)	−0.229 (−0.696, 0.238)	−0.064 (−0.527, 0.399)
Saxony-Anhalt (1)	−0.481 (−1.063, 0.102)	−0.437 (−1.016, 0.141)	−0.232 (−0.805, 0.341)
Schleswig-Holzstein (1)	−0.179 (−0.602, 0.243)	−0.263 (−0.683, 0.156)	−0.161 (−0.579, 0.257)
Thuringia (1)	−0.130 (−0.731, 0.471)	−0.153 (−0.750, 0.444)	−0.135 (−0.724, 0.455)
Female (2)	0.518*** (0.335, 0.700)	0.650*** (0.463, 0.836)	0.502*** (0.310, 0.694)
Age	0.256*** (0.154, 0.357)	0.278*** (0.177, 0.379)	0.205*** (0.102, 0.309)
Medium level of education (3)	−0.079 (−0.299, 0.141)	−0.079 (−0.298, 0.140)	0.081 (−0.139, 0.300)
High level of education (3)	−0.137 (−0.366, 0.092)	−0.148 (−0.376, 0.080)	0.114 (−0.123, 0.352)
Afghan (4)	0.776*** (0.506, 1.046)	0.807*** (0.534, 1.080)	0.551*** (0.267, 0.836)
Iraqi (4)	0.232 (−0.065, 0.530)	0.249* (−0.047, 0.544)	0.013 (−0.288, 0.314)
Eritrean (4)	−0.920*** (−1.371, −0.468)	−1.065*** (−1.521, −0.608)	−1.273*** (−1.724, −0.821)
Other (4)	0.710*** (0.469, 0.952)	0.857*** (0.609, 1.104)	0.496*** (0.225, 0.767)
Christian (5)	0.043 (−0.237, 0.323)	0.109 (−0.170, 0.388)	0.166 (−0.110, 0.442)
Other religious affiliation (5)	−0.200 (−0.578, 0.178)	−0.130 (−0.507, 0.247)	−0.149 (−0.520, 0.222)
No religious affiliation (5)	0.168 (−0.175, 0.511)	0.173 (−0.168, 0.514)	0.203 (−0.133, 0.538)
Single (6)	0.320*** (0.090, 0.551)	0.266** (0.029, 0.503)	0.496*** (0.246, 0.746)
Divorced/Widowed (6)	0.564*** (0.136, 0.992)	0.537** (0.112, 0.962)	0.615*** (0.193, 1.037)
Time since arrival	−0.114** (−0.203, −0.024)	−0.103** (−0.192, −0.014)	0.089* (−0.006, 0.184)
Number of flight reasons		0.203*** (0.114, 0.291)	0.203*** (0.117, 0.290)
Arrived in Germany alone (7)		0.197* (−0.019, 0.413)	−0.027 (−0.247, 0.194)
Negative flight experiences (8)		0.651*** (0.444, 0.857)	0.652*** (0.450, 0.855)
No answers on flight experience (8)		0.189* (−0.022, 0.400)	0.134 (−0.074, 0.341)
Subsidiary protection (9)			0.493** (0.021, 0.965)
Suspension of deportation (9)			0.749** (0.137, 1.362)
Awaiting outcome (9)			0.495*** (0.288, 0.702)
Seeking family reunification (10)			1.111*** (0.805, 1.417)
Private accommodation (11)			−0.446*** (−0.658, −0.233)
Currently in education (12)			0.324* (−0.049, 0.697)
Currently working (13)			−0.422*** (−0.710, −0.134)
Not seeking work (13)			0.312* (−0.016, 0.640)
Number of courses attended			−0.013 (−0.105, 0.079)
Time with people from count. o. origin			−0.079* (−0.172, 0.013)
Time with Germans			−0.176*** (−0.270, −0.082)
Time with others			−0.077 (−0.171, 0.016)
German language ability			−0.156*** (−0.261, −0.052)
Observations	4,325	4,325	4,325
R^2	0.051	0.067	0.107
Adjusted R^2	0.045	0.06	0.098
$\Delta Adjusted R^2$		0.015***	0.038***
Note:			*p<0.1; **p<0.05; ***p<0.01

Table A4. Predicting Psychological Distress, Imputed Data. Regression Coefficients with 95%-confidence intervals as plotted in Figure 1. Hierarchical regressions comprising three models each (Model 1a-3a and Model 1b-3b). Regression coefficient estimates pooled across 10 imputed datasets. Predictor variables are standardized for comparison purposes. Reference category for (1) = Badem-Württemberg; (2) = Male, (3) = Low level of education, (4) = Syrian, (5) = Muslim, (6) = Married, (7) = Did not arrive alone, (8) = No negative flight experiences, (9) = Refugee status or asylum, (10) = Not seeking, (11) = Refugee housing facility, (12) = Currently not in education, (13) = Currently not working.

	Dependent variable:		
	Psychological Distress		
	Model 1a	Model 2a	Model 3a
	Beta Coef. (95%-CI)	Beta Coef. (95%-CI)	Beta Coef. (95%-CI)
Intercept	2.838*** (2.540, 3.135)	2.416*** (2.092, 2.740)	2.538*** (2.165, 2.912)
Bavaria (1)	−0.381** (−0.723, −0.039)	−0.331* (−0.673, 0.011)	−0.279 (−0.619, 0.060)
Berlin (1)	0.123 (−0.320, 0.566)	0.147 (−0.293, 0.587)	−0.109 (−0.546, 0.329)
Brandenburg (1)	−0.101 (−0.602, 0.401)	−0.100 (−0.597, 0.398)	−0.076 (−0.566, 0.414)
Bremen (1)	−0.614 (−1.432, 0.204)	−0.479 (−1.293, 0.335)	−0.251 (−1.060, 0.557)
Hamburg (1)	−1.064*** (−1.774, −0.354)	−0.929*** (−1.635, −0.223)	−1.054*** (−1.750, −0.357)
Hesse (1)	−0.570** (−1.004, −0.136)	−0.473** (−0.909, −0.038)	−0.401* (−0.831, 0.028)
Meckelenburg-Vorpommern (1)	−0.366 (−1.090, 0.359)	−0.181 (−0.903, 0.541)	−0.023 (−0.734, 0.687)
Lower Saxony (1)	−0.353* (−0.755, 0.048)	−0.363* (−0.762, 0.036)	−0.123 (−0.522, 0.275)
North Rhine-Westphalia (1)	−0.146 (−0.481, 0.190)	−0.052 (−0.389, 0.286)	0.082 (−0.257, 0.420)
Rhineland-Palatinate (1)	0.956*** (0.405, 1.507)	0.903*** (0.356, 1.450)	1.059*** (0.517, 1.602)
Saarland (1)	−0.191 (−0.758, 0.376)	−0.214 (−0.778, 0.349)	0.094 (−0.468, 0.656)
Saxony (1)	−0.248 (−0.772, 0.276)	−0.162 (−0.683, 0.359)	0.025 (−0.492, 0.543)
Saxony-Anhalt (1)	−0.469 (−1.126, 0.187)	−0.402 (−1.054, 0.251)	−0.183 (−0.829, 0.463)
Schleswig-Holzstein (1)	−0.071 (−0.537, 0.394)	−0.148 (−0.610, 0.315)	−0.062 (−0.522, 0.397)
Thuringia (1)	0.005 (−0.696, 0.706)	−0.044 (−0.741, 0.653)	−0.006 (−0.694, 0.683)
Female (2)	0.525*** (0.318, 0.731)	0.659*** (0.447, 0.872)	0.517*** (0.298, 0.735)
Age	0.289*** (0.172, 0.406)	0.310*** (0.194, 0.426)	0.244*** (0.126, 0.362)
Medium level of education (3)	−0.124 (−0.364, 0.115)	−0.127 (−0.365, 0.111)	0.043 (−0.197, 0.283)
High level of education (3)	−0.125 (−0.374, 0.124)	−0.129 (−0.377, 0.118)	0.129 (−0.131, 0.388)
Afghan (4)	0.790*** (0.476, 1.104)	0.832*** (0.514, 1.150)	0.599*** (0.269, 0.930)
Iraqi (4)	0.223 (−0.103, 0.549)	0.243 (−0.081, 0.567)	0.023 (−0.308, 0.353)
Eritrean (4)	−1.012*** (−1.529, −0.495)	−1.142*** (−1.667, −0.618)	−1.359*** (−1.877, −0.841)
Other (4)	0.808*** (0.536, 1.080)	0.966*** (0.689, 1.243)	0.633*** (0.330, 0.935)
Christian (5)	0.018 (−0.296, 0.332)	0.083 (−0.231, 0.396)	0.141 (−0.168, 0.449)
Other religious affiliation (5)	−0.102 (−0.517, 0.312)	−0.044 (−0.457, 0.369)	−0.055 (−0.461, 0.350)
No religious affiliation (5)	0.191 (−0.200, 0.582)	0.198 (−0.191, 0.587)	0.226 (−0.155, 0.608)
Single (6)	0.290** (0.030, 0.550)	0.252* (−0.016, 0.519)	0.495*** (0.214, 0.776)
Divorced/Widowed (6)	0.447* (−0.053, 0.947)	0.438* (−0.058, 0.934)	0.592** (0.105, 1.079)
Time since arrival	−0.136*** (−0.239, −0.034)	−0.131** (−0.233, −0.029)	0.097* (−0.013, 0.207)
Number of flight reasons		0.201*** (0.101, 0.300)	0.198*** (0.100, 0.296)
Arrived in Germany alone (7)		0.155 (−0.092, 0.402)	−0.104 (−0.353, 0.146)
Negative flight experiences (8)		0.623*** (0.393, 0.852)	0.649*** (0.423, 0.875)
No answers on flight experience (8)		0.116 (−0.126, 0.357)	0.082 (−0.155, 0.318)
Subsidiary protection (9)			0.553** (0.015, 1.091)
Suspension of deportation (9)			0.569* (−0.101, 1.238)
Awaiting outcome (9)			0.436*** (0.207, 0.664)
Seeking family reunification (10)			1.184*** (0.842, 1.526)
Private accommodation (11)			−0.614*** (−0.852, −0.376)
Currently in education (12)			0.294 (−0.135, 0.723)
Currently working (13)			−0.467*** (−0.790, −0.144)
Not seeking work (13)			0.297 (−0.073, 0.666)
Number of courses attended			−0.002 (−0.105, 0.102)
Time with people from count. o. origin			−0.079 (−0.181, 0.023)
Time with Germans			−0.194*** (−0.298, −0.090)
Time with others			−0.085 (−0.189, 0.018)
German language ability			−0.144** (−0.263, −0.025)
Observations	3,303	3,303	3,303
R^2	0.056	0.072	0.116
Adjusted R^2	0.048	0.063	0.104
$\Delta Adjusted R^2$		0.015***	0.041***
Note:			*p<0.1; **p<0.05; ***p<0.01

Table A5. Predicting Psychological, Unimputed Data. Regression Coefficients with 95%-confidence Predictor variables are standardized for comparison purposes. Reference category for (1) = Badem-Württemberg; (2) = Male, (3) = Low level of education, (4) = Syrian, (5) = Muslim, (6) = Married, (7) = Did not arrive alone, (8) = No negative flight experiences, (9) = Refugee status or asylum, (10) = Not seeking, (11) = Refugee housing facility, (12) = Currently not in education, (13) = Currently not working.

	Dependent variable:		
	Life Satisfaction		
	Model 1b	Model 2b	Model 3b
	Beta Coef. (95%-CI)	Beta Coef. (95%-CI)	Beta Coef. (95%-CI)
Intercept	7.456*** (7.232, 7.679)	7.747*** (7.504, 7.989)	7.593*** (7.314, 7.872)
Bavaria (1)	0.042 (−0.216, 0.299)	0.092 (−0.167, 0.350)	0.011 (−0.243, 0.265)
Berlin (1)	−0.545*** (−0.867, −0.223)	−0.533*** (−0.854, −0.211)	−0.334** (−0.652, −0.016)
Brandenburg (1)	−0.228 (−0.592, 0.136)	−0.244 (−0.606, 0.119)	−0.268 (−0.626, 0.090)
Bremen (1)	0.205 (−0.433, 0.843)	0.082 (−0.555, 0.718)	−0.195 (−0.824, 0.433)
Hamburg (1)	−0.174 (−0.673, 0.326)	−0.207 (−0.705, 0.291)	−0.131 (−0.619, 0.358)
Hesse (1)	0.057 (−0.262, 0.376)	0.108 (−0.213, 0.428)	−0.016 (−0.331, 0.298)
Meckelenburg-Vorpommern (1)	0.308 (−0.214, 0.830)	0.287 (−0.234, 0.808)	0.068 (−0.442, 0.579)
Lower Saxony (1)	0.038 (−0.263, 0.340)	0.101 (−0.201, 0.402)	−0.206 (−0.505, 0.093)
North Rhine-Westphalia (1)	0.229* (−0.017, 0.476)	0.272** (0.022, 0.521)	0.081 (−0.168, 0.329)
Rhineland-Palatinate (1)	−0.491** (−0.914, −0.068)	−0.462** (−0.884, −0.041)	−0.709*** (−1.124, −0.294)
Saarland (1)	0.297 (−0.126, 0.719)	0.353 (−0.068, 0.775)	−0.034 (−0.452, 0.385)
Saxony (1)	−0.342* (−0.729, 0.045)	−0.340* (−0.726, 0.046)	−0.579*** (−0.959, −0.198)
Saxony-Anhalt (1)	0.184 (−0.304, 0.671)	0.171 (−0.315, 0.657)	−0.085 (−0.563, 0.394)
Schleswig-Holzstein (1)	−0.001 (−0.355, 0.353)	0.059 (−0.294, 0.412)	−0.143 (−0.492, 0.206)
Thuringia (1)	−0.848*** (−1.351, −0.346)	−0.870*** (−1.371, −0.370)	−0.925*** (−1.417, −0.433)
Female (2)	0.241*** (0.090, 0.392)	0.165** (0.009, 0.320)	0.204** (0.044, 0.364)
Age	−0.145*** (−0.230, −0.059)	−0.148*** (−0.233, −0.063)	−0.116*** (−0.202, −0.030)
Medium level of education (3)	−0.119 (−0.298, 0.060)	−0.130 (−0.308, 0.048)	−0.243*** (−0.421, −0.065)
High level of education (3)	−0.225** (−0.417, −0.032)	−0.239** (−0.431, −0.047)	−0.467*** (−0.666, −0.269)
Afghan (4)	0.300*** (0.077, 0.523)	0.295** (0.069, 0.521)	0.563*** (0.327, 0.798)
Iraqi (4)	−0.152 (−0.397, 0.093)	−0.152 (−0.396, 0.092)	0.089 (−0.158, 0.337)
Eritrean (4)	0.293 (−0.085, 0.670)	0.455** (0.073, 0.838)	0.740*** (0.363, 1.116)
Other (4)	−0.157 (−0.355, 0.041)	−0.220** (−0.422, −0.019)	0.137 (−0.085, 0.358)
Christian (5)	−0.259** (−0.496, −0.023)	−0.312*** (−0.548, −0.076)	−0.363*** (−0.593, −0.132)
Other religious affiliation (5)	0.240 (−0.066, 0.547)	0.212 (−0.094, 0.518)	0.223 (−0.076, 0.521)
No religious affiliation (5)	0.199 (−0.089, 0.487)	0.211 (−0.075, 0.498)	0.158 (−0.123, 0.438)
Single (6)	−0.550*** (−0.742, −0.357)	−0.456*** (−0.655, −0.257)	−0.597*** (−0.805, −0.388)
Divorced/Widowed (6)	−0.798*** (−1.147, −0.450)	−0.773*** (−1.120, −0.425)	−0.794*** (−1.133, −0.455)
Time since arrival	0.159*** (0.085, 0.234)	0.157*** (0.083, 0.232)	−0.041 (−0.119, 0.038)
Number of flight reasons		−0.126*** (−0.199, −0.053)	−0.124*** (−0.195, −0.053)
Arrived in Germany alone (7)		−0.289*** (−0.470, −0.108)	−0.044 (−0.227, 0.139)
Negative flight experiences (8)		−0.298*** (−0.471, −0.125)	−0.290*** (−0.460, −0.121)
No answers on flight experience (8)		−0.393*** (−0.568, −0.219)	−0.359*** (−0.530, −0.189)
Subsidiary protection (9)			−0.299 (−0.693, 0.095)
Suspension of deportation (9)			−0.303 (−0.804, 0.197)
Awaiting outcome (9)			−0.453*** (−0.621, −0.286)
Seeking family reunification (10)			−0.860*** (−1.119, −0.601)
Private accommodation (11)			0.614*** (0.439, 0.789)
Currently in education (12)			−0.066 (−0.377, 0.246)
Currently working (13)			0.186 (−0.050, 0.422)
Not seeking work (13)			0.107 (−0.165, 0.380)
Number of courses attended			0.002 (−0.074, 0.078)
Time with people from count. o. origin			−0.023 (−0.098, 0.052)
Time with Germans			0.247*** (0.170, 0.323)
Time with others			0.006 (−0.071, 0.082)
German language ability			0.121*** (0.033, 0.208)
Observations	4,325	4,325	4,325
R^2	0.043	0.053	0.105
Adjusted R^2	0.036	0.045	0.095
$\Delta Adjusted R^2$		0.009***	0.05***
Note:			*p<0.1; **p<0.05; ***p<0.01

Table A6. Predicting Life Satisfaction, Imputed Data. Regression Coefficients with 95%-confidence intervals as plotted in Figure 1, Model 1b-3b. Regression coefficient estimates pooled across 10 imputed datasets. Predictor variables are standardized for comparison purposes. Reference category for (1) = Badem-Württemberg; (2) = Male, (3) = Low level of education, (4) = Syrian, (5) = Muslim, (6) = Married, (7) = Did not arrive alone, (8) = No negative flight experiences, (9) = Refugee status or asylum, (10) = Not seeking, (11) = Refugee housing facility, (12) = Currently not in education, (13) = Currently not working.

	Dependent variable:		
		Life Satisfaction	
	(Model 1b)	(Model 2b)	(Model 3b)
Intercept	7.505*** (7.265, 7.745)	7.793*** (7.532, 8.055)	7.687*** (7.387, 7.986)
Bavaria (1)	0.049 (−0.229, 0.326)	0.078 (−0.200, 0.357)	−0.023 (−0.297, 0.250)
Berlin (1)	−0.700*** (−1.057, −0.343)	−0.684*** (−1.040, −0.328)	−0.413** (−0.764, −0.062)
Brandenburg (1)	−0.205 (−0.609, 0.200)	−0.218 (−0.621, 0.184)	−0.243 (−0.637, 0.151)
Bremen (1)	0.218 (−0.430, 0.866)	0.103 (−0.544, 0.749)	−0.151 (−0.790, 0.488)
Hamburg (1)	−0.126 (−0.682, 0.430)	−0.207 (−0.762, 0.348)	−0.105 (−0.648, 0.437)
Hesse (1)	0.186 (−0.165, 0.536)	0.221 (−0.131, 0.573)	0.100 (−0.245, 0.445)
Meckelenburg-Vorpommern (1)	0.595** (0.024, 1.167)	0.542* (−0.029, 1.113)	0.359 (−0.199, 0.917)
Lower Saxony (1)	−0.039 (−0.356, 0.277)	0.006 (−0.310, 0.323)	−0.268* (−0.581, 0.046)
North Rhine-Westphalia (1)	0.213 (−0.056, 0.483)	0.240* (−0.032, 0.512)	0.061 (−0.210, 0.332)
Rhineland-Palatinate (1)	−0.615*** (−1.061, −0.169)	−0.575** (−1.020, −0.131)	−0.786*** (−1.224, −0.349)
Saarland (1)	0.307 (−0.144, 0.758)	0.364 (−0.086, 0.814)	−0.016 (−0.462, 0.430)
Saxony (1)	−0.291 (−0.708, 0.126)	−0.300 (−0.716, 0.117)	−0.532** (−0.942, −0.122)
Saxony-Anhalt (1)	0.086 (−0.442, 0.615)	0.060 (−0.466, 0.587)	−0.206 (−0.724, 0.312)
Schleswig-Holzstein (1)	−0.021 (−0.400, 0.357)	0.034 (−0.343, 0.412)	−0.118 (−0.489, 0.254)
Thuringia (1)	−0.913*** (−1.446, −0.380)	−0.918*** (−1.450, −0.387)	−0.947*** (−1.469, −0.425)
Female (2)	0.235*** (0.070, 0.399)	0.174** (0.004, 0.344)	0.208** (0.034, 0.382)
Age	−0.096** (−0.189, −0.002)	−0.104** (−0.197, −0.010)	−0.071 (−0.165, 0.024)
Medium level of education (3)	−0.169* (−0.362, 0.024)	−0.169* (−0.361, 0.024)	−0.273*** (−0.465, −0.081)
High level of education (3)	−0.260** (−0.461, −0.059)	−0.273*** (−0.473, −0.072)	−0.485*** (−0.692, −0.277)
Afghan (4)	0.331*** (0.082, 0.579)	0.312** (0.059, 0.565)	0.581*** (0.319, 0.842)
Iraqi (4)	−0.004 (−0.266, 0.257)	−0.006 (−0.267, 0.256)	0.244* (−0.020, 0.508)
Eritrean (4)	0.254 (−0.151, 0.659)	0.382* (−0.030, 0.794)	0.687*** (0.282, 1.092)
Other (4)	−0.186* (−0.404, 0.032)	−0.271** (−0.493, −0.048)	0.116 (−0.127, 0.359)
Christian (5)	−0.295** (−0.548, −0.043)	−0.353*** (−0.605, −0.100)	−0.414*** (−0.660, −0.167)
Other religious affiliation (5)	0.182 (−0.144, 0.509)	0.159 (−0.168, 0.486)	0.158 (−0.161, 0.477)
No religious affiliation (5)	0.113 (−0.200, 0.427)	0.123 (−0.190, 0.435)	0.068 (−0.237, 0.372)
Single (6)	−0.475*** (−0.683, −0.267)	−0.425*** (−0.641, −0.210)	−0.598*** (−0.823, −0.374)
Divorced/Widowed (6)	−0.841*** (−1.240, −0.442)	−0.837*** (−1.235, −0.440)	−0.892*** (−1.280, −0.504)
Time since arrival	0.159*** (0.077, 0.242)	0.156*** (0.074, 0.238)	−0.056 (−0.143, 0.032)
Number of flight reasons		−0.137*** (−0.217, −0.057)	−0.133*** (−0.211, −0.055)
Arrived in Germany alone (7)		−0.148 (−0.347, 0.051)	0.112 (−0.088, 0.312)
Negative flight experiences (8)		−0.342*** (−0.527, −0.158)	−0.341*** (−0.521, −0.160)
No answers on flight experience (8)		−0.381*** (−0.574, −0.189)	−0.362*** (−0.550, −0.174)
Subsidiary protection (9)			−0.472** (−0.906, −0.039)
Suspension of deportation (9)			−0.460* (−1.007, 0.088)
Awaiting outcome (9)			−0.506*** (−0.688, −0.324)
Seeking family reunification (10)			−0.976*** (−1.247, −0.704)
Private accommodation (11)			0.595*** (0.405, 0.785)
Currently in education (12)			−0.045 (−0.389, 0.299)
Currently working (13)			0.127 (−0.132, 0.386)
Not seeking work (13)			0.054 (−0.241, 0.350)
Number of courses attended			−0.019 (−0.102, 0.064)
Time with people from count. o. origin			−0.023 (−0.104, 0.059)
Time with Germans			0.260*** (0.177, 0.343)
Time with others			0.007 (−0.076, 0.089)
German language ability			0.131*** (0.036, 0.225)
Observations	3,549	3,549	3,549
R^2	0.046	0.056	0.113
Adjusted R^2	0.038	0.047	0.101
ΔR^2		0.009***	0.054***
Note:			*p<0.1; **p<0.05; ***p<0.01

Table A7. Predicting Life Satisfaction, Unimputed Data. Regression Coefficients with 95%-confidence. Predictor variables are standardized for comparison purposes. Reference category for (1) = Badem-Württemberg; (2) = Male, (3) = Low level of education, (4) = Syrian, (5) = Muslim, (6) = Married, (7) = Did not arrive alone, (8) = No negative flight experiences, (9) = Refugee status or asylum, (10) = Not seeking, (11) = Refugee housing facility, (12) = Currently not in education, (13) = Currently not working.

	Dependent variable:
	Psychological Distress, Ordinal
None—Mild Distress	0.238* (−0.006, 0.481)
Mild—Moderate Distress	2.010*** (1.757, 2.263)
Moderate—Severe Distress	3.368*** (3.093, 3.643)
Bavaria (1)	−0.252** (−0.478, −0.027)
Berlin (1)	−0.233* (−0.510, 0.043)
Brandenburg (1)	−0.016 (−0.320, 0.287)
Bremen (1)	−0.080 (−0.586, 0.426)
Hamburg (1)	−0.377* (−0.796, 0.041)
Hesse (1)	−0.352** (−0.635, −0.069)
Meckelenburg-Vorpommern (1)	0.322 (−0.106, 0.751)
Lower Saxony (1)	−0.169 (−0.436, 0.098)
North Rhine-Westphalia (1)	−0.144 (−0.363, 0.075)
Rhineland-Palatinate (1)	0.548*** (0.199, 0.896)
Saarland (1)	−0.036 (−0.408, 0.336)
Saxony (1)	−0.004 (−0.335, 0.328)
Saxony-Anhalt (1)	−0.135 (−0.560, 0.290)
Schleswig-Holzstein (1)	−0.057 (−0.357, 0.243)
Thuringia (1)	−0.043 (−0.472, 0.387)
Female (2)	0.353*** (0.215, 0.491)
Age	0.137*** (0.062, 0.211)
Medium level of education (3)	0.045 (−0.118, 0.207)
High level of education (3)	0.108 (−0.066, 0.282)
Afghanistan (4)	0.316*** (0.108, 0.525)
Iraq (4)	−0.016 (−0.237, 0.205)
Eritrea (4)	−0.894*** (−1.249, −0.538)
Other (4)	0.284*** (0.088, 0.480)
Christian (5)	0.052 (−0.153, 0.256)
Other religious affiliation (5)	−0.106 (−0.378, 0.165)
No religious affiliation (5)	0.075 (−0.173, 0.322)
Single (6)	0.276*** (0.093, 0.459)
Divorced/Widowed (6)	0.306** (0.003, 0.609)
Time since arrival	0.062* (−0.007, 0.132)
Number of flight reasons	0.136*** (0.072, 0.199)
Arrived in Germany alone (7)	−0.025 (−0.186, 0.136)
Negative flight experiences (8)	0.413*** (0.264, 0.562)
No answers on flight experience (8)	0.139* (−0.013, 0.292)
Subsidiary protection (9)	0.352** (0.018, 0.686)
Suspension of deportation (9)	0.511** (0.078, 0.944)
Awaiting outcome (9)	0.394*** (0.247, 0.541)
Seeking family reunification (10)	0.673*** (0.453, 0.892)
Private accommodation (11)	−0.333*** (−0.485, −0.181)
Currently in education (12)	0.278** (0.002, 0.554)
Currently working (13)	−0.351*** (−0.569, −0.133)
Not seeking work (13)	0.195 (−0.041, 0.432)
Number of courses attended	0.002 (−0.064, 0.068)
Time with people from count. o. origin	−0.013 (−0.079, 0.053)
Time with Germans	−0.136*** (−0.205, −0.068)
Time with others	−0.045 (−0.113, 0.022)
German language ability	−0.090** (−0.167, −0.013)
Observations	4,325

Note:
*p<0.1; **p<0.05; ***p<0.01

Table A8. Predicting Psychological Distress, Cumulative Link Model. Regression Coefficients with 95%-confidence intervals, Model 3a with psychological distress as an ordinal outcome variable. Regression coefficient estimates pooled across 10 imputed datasets. Predictor variables are standardized for comparison purposes. Reference category for (1) = Badem-Württemberg; (2) = Male, (3) = Low level of education, (4) = Syrian, (5) = Muslim, (6) = Married, (7) = Did not arrive alone, (8) = No negative flight experiences, (9) = Refugee status or asylum, (10) = Not seeking, (11) = Refugee housing facility, (12) = Currently not in education, (13) = Currently not working.
